# Supplementary material for: Ferrotoroidic ground state in a heterometallic {CrIIIDyIII6} complex displaying slow magnetic relaxation
Source: Nat Commun. 2017 Oct 18;8:1023. doi: 10.1038/s41467-017-01102-5 (PMC5647347; doi:10.1038/s41467-017-01102-5)
Supplement: Supplementary file 1 — Supplementary Information [file 41467_2017_1102_MOESM1_ESM.pdf]

Supplementary Methods | **Reaction scheme used to isolate compound **1.

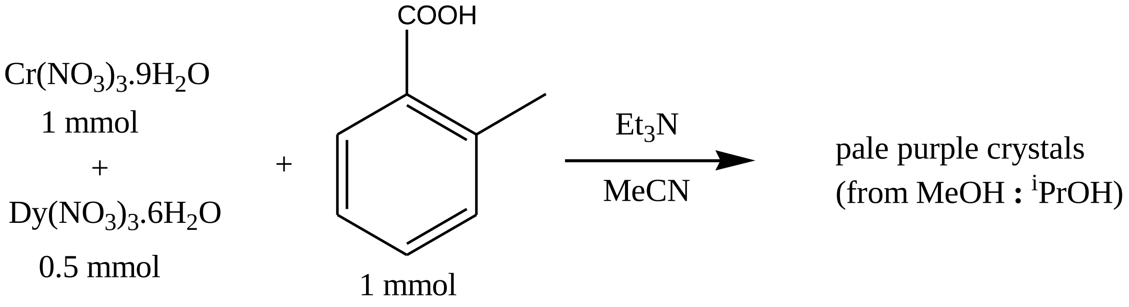

Supplementary Table 1. X-ray crystallographic data for **1**.

	**1**
Formula[a]	$CrDy_6C_{104}H_{124}NO_{43}$
M, $gmol^{-1}$	3075
Crystal system	Triclinic
Space group	P-1
a/Å	14.970(3)
b/Å	15.013(3)
c/Å	16.381(3)
α/deg	63.49(3)
β/deg	68.16(3)
γ/deg	68.27(3)
V/$Å^3$	2964.1(10)
T/K	100(2)
Z	1
$\rho_{, calc}$ [g cm^{-3}]	1.738
λ[b]/ Å	0.71079
Data Measured	64494
Ind. Reflns	13680
R_{int}	0.0539
Reflns with I<br>I > 2σ(I)	13545
Parameters	769
Restraints	65
R_1[c] (I > 2σ(I)), wR_2[c] (all data)	0.0346, 0.0866
goodness of fit	1.126
Largest residuals/e $Å^{-3}$	1.656, -1.608

Supplementary Table 2. Selected bond lengths for compound **1**. (See figure 1a for labelling)

Atoms- Bond lengths (Å)			
Dy1 O2 - 2.286(3)	Dy2 O15 - 2.316(3)	Dy3 O35 - 2.249(4)	Cr1 $O5^{I}$ – 1.970(2)
Dy1 O9 - 2.303(3)	Dy2 O12 - 2.323(3)	Dy3 O14 - 2.313(3)	Cr1 O5 – 1.970(2)
Dy1 O3 - 2.357(3)	Dy2 O3 - 2.356(3)	Dy3 O18 - 2.354(6)	Cr1 O1 – 1.981(3)
Dy1 O6 - 2.379(3)	Dy2 O5 - 2.370(3)	Dy3 O21 - 2.366(5)	Cr1 $O1^{I}$ – 1.981(3)
Dy1 O11 - 2.383(2)	Dy2 O16 - 2.396(3)	Dy3 O1 - 2.370(3)	Cr1 $O11^{I}$ – 1.983(3)
Dy1 O7 - 2.391(3)	Dy2 O4 - 2.398(3)	Dy3 O3 - 2.372(3)	Cr1 O11 – 1.983(3)
Dy1 O10 - 2.416(3)	Dy2 O8 - 2.418(3)	Dy3 O13 - 2.420(3)	Dy1 Dy2 - 3.750
Dy1 O5 - 2.433(3)	Dy2 O1 - 2.436(2)	Dy3 O11 - 2.426(3)	Dy2Dy3- 3.767
		Dy3O20 – 2.492(15)	Dy1Dy3- 3.780
Atoms- Bond angles(^{o})			
Dy1O3Dy2 -105.1 ^{o}	Dy1O5Dy2 -104.2 ^{o}	Cr1O5Dy1 - 102.9 ^{o}	Cr1O1Dy2 - 102.9 ^{o}
Dy1O3Dy3 -106.6 ^{o}	Dy1O11Dy3 -102.6 ^{o}	Cr1O11Dy1 -105.0 ^{o}	Cr1O1Dy3 - 105.6 ^{o}
Dy2O3Dy3 -106.3 ^{o}	Dy2O1Dy3 -103.3 ^{o}	Cr1O5Dy2 - 104.3 ^{o}	Cr1O11Dy3 -103.0 ^{o}
**Symmetry transformation; ^{I} 1-X, -Y, 1-Z.**			

Supplementary Table 3. *Ab initio* Computed Ground-State g-Tensors for each Dy center and Cr in **1**.

	Dy1	Dy2	Dy3	Dy1'	Dy2'	Dy3'	Cr
g_x	0.0523	0.0737	0.0233	0.1633	0.0859	0.0203	2.0023
g_y	0.0917	0.0979	0.0361	0.3481	0.1006	0.0356	2.0023
g_z	19.5707	19.4723	19.6059	19.2240	19.4648	19.6217	2.0023

Supplementary Table 4. Energies of the Lowest Kramer's Doublet (KDs) of each Dy Center in **1**

KDs	E(cm^{-1})					
	Dy1	Dy2	Dy3	Dy1'	Dy2'	Dy3'
1	0.000	0.000	0.000	0.000	0.000	0.000
2	142.839	121.911	152.746	94.096	117.124	151.782
3	250.684	175.149	242.427	149.810	147.665	238.221
4	278.388	200.238	275.561	223.345	178.538	261.857
5	320.341	235.719	303.708	262.081	224.639	292.470
6	386.023	277.055	342.999	325.666	275.750	346.059
7	417.227	318.409	402.913	391.078	301.710	382.199
8	598.113	507.161	526.648	596.385	485.772	522.690

Supplementary Table 5. RASSI energies of the lowest spin-orbit states (cm^{-1}) on each Dy center in complex **1**.

Dy1	Dy2	Dy3	Dy1'	Dy2'	Dy3'
0.000	0.000	0.000	0.000	0.000	0.000
142.839	121.911	152.746	94.096	117.124	151.782
250.684	175.149	242.427	149.810	147.665	238.221
278.388	200.238	275.561	223.345	178.538	261.857
320.341	235.719	303.708	262.081	224.639	292.470
386.023	277.055	342.999	325.666	275.750	346.059
417.227	318.409	402.913	391.078	301.710	382.199
598.113	507.161	526.648	596.385	485.772	522.690
3064.645	3054.606	3074.156	3050.378	3051.190	3071.004
3175.816	3126.183	3174.608	3118.981	3113.213	3175.592
3245.406	3165.332	3218.460	3167.958	3149.631	3214.018
3279.445	3193.872	3258.765	3205.075	3185.583	3250.228
3310.145	3233.799	3294.038	3260.013	3223.063	3287.395
3347.817	3267.797	3337.611	3346.586	3252.106	3324.939
3465.300	3357.391	3400.436	3443.568	3333.258	3390.684
5678.787	5660.257	5697.522	5649.267	5652.365	5691.543
5771.359	5699.632	5749.368	5701.544	5684.735	5745.745
5816.455	5744.679	5791.126	5760.421	5738.326	5786.778
5866.429	5789.886	5843.186	5793.518	5776.681	5838.672
5910.047	5837.681	5897.265	5908.725	5827.193	5892.065
6002.422	5896.043	5955.957	5978.470	5870.996	5941.873
7881.448	7846.890	7895.647	7835.582	7833.906	7885.779
7951.903	7890.696	7933.847	7892.077	7880.401	7929.269
8010.601	7930.615	7977.075	7944.348	7922.663	7978.407
8069.725	7998.624	8050.248	8057.531	7987.233	8044.634
8160.215	8062.117	8126.159	8131.445	8039.733	8113.269
9615.778	9566.384	9604.569	9564.374	9558.664	9602.403
9659.671	9603.227	9652.642	9615.078	9588.390	9642.564
9699.321	9632.596	9689.991	9641.403	9616.922	9682.620
9720.235	9649.181	9711.924	9667.293	9635.200	9702.083
9746.063	9676.165	9724.393	9699.030	9664.445	9717.041
9764.997	9687.892	9739.871	9723.434	9673.999	9734.785
9780.431	9713.287	9759.978	9757.407	9702.807	9756.172
9817.463	9742.849	9782.707	9793.183	9733.159	9782.209
9858.276	9777.402	9827.070	9829.222	9764.845	9822.335
9960.088	9859.809	9923.271	9933.927	9839.773	9912.372
11033.571	11000.679	11036.767	10998.193	10994.377	11035.968
11196.953	11106.654	11180.450	11120.425	11091.518	11169.149
11334.515	11245.606	11289.242	11321.904	11227.435	11282.283
11861.748	11792.368	11846.713	11811.576	11779.775	11839.546
11902.901	11841.188	11886.334	11869.016	11826.413	11879.797
11928.297	11847.554	11899.434	11896.181	11834.777	11893.337
11939.214	11864.172	11909.833	11909.159	11853.166	11906.234
11979.201	11898.279	11954.724	11941.381	11884.328	11948.386
13638.669	13572.873	13624.016	13601.627	13559.052	13616.914
13674.222	13594.818	13646.290	13630.281	13582.027	13640.367

13734.400	13661.189	13709.497	13693.054	13648.660	13704.201
13748.747	13675.642	13726.917	13720.403	13662.561	13721.190
15047.530	14974.830	15024.607	15008.672	14961.272	15018.016
15083.585	15009.079	15062.187	15047.116	14996.104	15056.314
15115.909	15045.029	15093.551	15077.880	15032.269	15087.866
16067.481	15996.096	16046.066	16026.753	15983.326	16040.107
16082.527	16008.426	16058.870	16047.196	15995.020	16052.782
16669.422	16595.597	16645.969	16631.710	16582.338	16639.881
38863.904	38833.695	38861.673	38819.623	38830.762	38863.948
38937.837	38904.879	38938.881	38923.555	38893.405	38933.232
39010.032	38958.100	38999.528	39002.845	38942.365	38991.282
39156.925	39037.093	39100.134	39090.914	39022.341	39095.137
40254.710	40221.513	40250.799	40217.950	40215.172	40249.859
40407.315	40321.102	40368.702	40339.587	40313.853	40369.508
40578.827	40467.096	40534.439	40578.249	40440.300	40517.090

Supplementary Table 6. The *g*-tensors for the eight lowest Kramer's doublets in **1**.

Kramer's doublet		Dy1	Dy2	Dy3	Dy1'	Dy2'	Dy3'
1	g_x	0.0523	0.0737	0.0233	0.1633	0.0859	0.0203
	g_y	0.0917	0.0979	0.0361	0.3481	0.1006	0.0356
	g_z	19.5707	19.4723	19.6059	19.2240	19.4648	19.6217
2	g_x	0.6831	0.8211	0.3570	0.8364	1.2016	0.5042
	g_y	0.8836	1.3395	0.4520	1.5092	1.8258	0.5767
	g_z	16.1314	15.5887	16.4132	15.4053	15.2248	16.3829
3	g_x	1.1077	0.1012	0.1905	0.0617	0.3731	0.4879
	g_y	1.9123	0.9926	0.3842	2.4196	1.1095	1.6275
	g_z	12.8316	17.5449	18.8231	12.4304	18.5865	17.2343
4	g_x	0.1593	2.8806	2.6274	1.8987	1.8359	1.6378
	g_y	1.6854	5.1992	4.8073	2.7027	4.5456	3.6599
	g_z	14.6884	12.7703	10.6397	14.2155	12.4248	11.9202
5	g_x	3.2954	9.2914	1.1275	7.7357	10.3423	7.9888
	g_y	4.3043	5.7892	5.0367	6.1907	6.2609	6.1581
	g_z	12.5431	0.4029	10.7298	1.1361	2.0397	1.1761
6	g_x	2.7288	2.0542	4.2213	2.3451	0.6258	3.0369
	g_y	3.6217	3.1486	4.9437	4.1734	2.8863	5.4057
	g_z	8.9464	14.7278	12.5575	13.9135	14.9129	12.5247
7	g_x	11.5008	0.2936	0.2485	0.6471	0.7873	0.8622
	g_y	7.2732	0.6093	0.3376	1.3238	1.6752	1.5110
	g_z	1.3588	16.6584	16.9158	15.4589	15.7159	15.8093
8	g_x	0.0094	0.0232	0.0443	0.0099	0.0227	0.0204
	g_y	0.0096	0.0351	0.0588	0.2079	0.0391	0.03071
	g_z	19.6474	19.6861	19.3823	19.3655	19.6361	19.4563

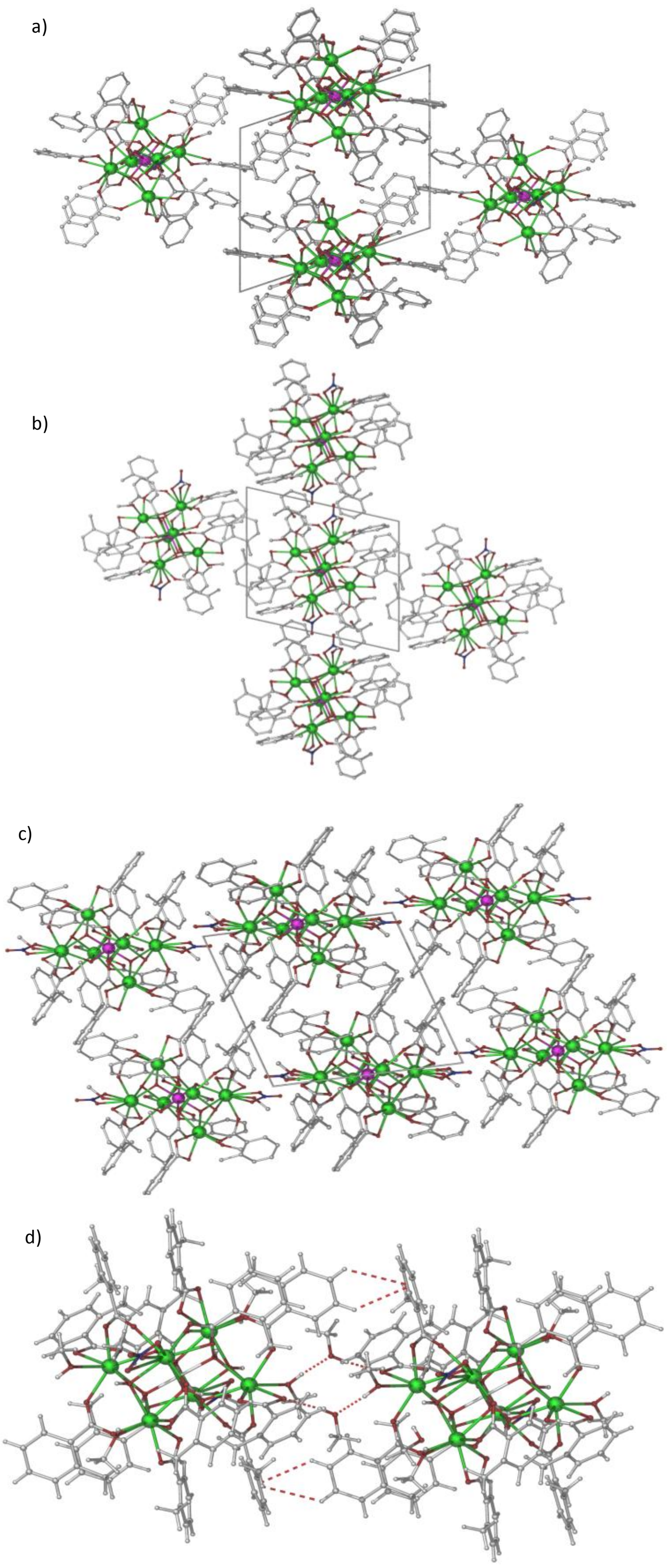

Supplementary Figure 1. Packing diagram of compound **1**, with views along the crystallographic a) *a* axis b) *b* axis, c) *c* axis and d) a highlights the intermolecular interactions between a neighbouring pair.

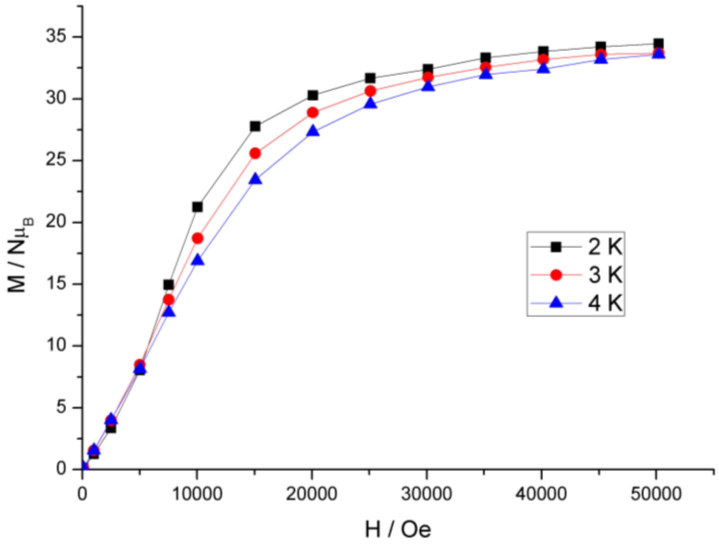

Supplementary Figure 2. Plots of (left) M versus H isotherms for complex **1** at 2, 3, and 4 K.

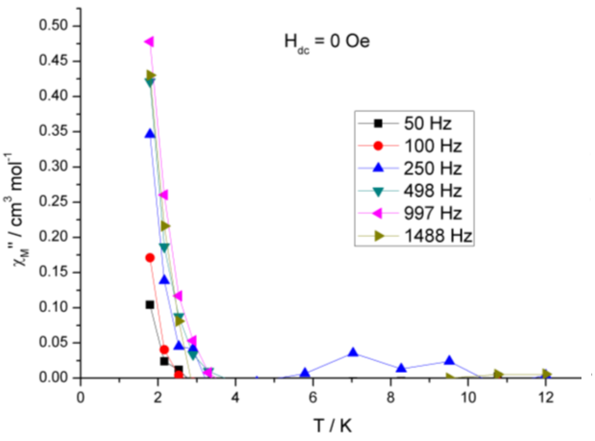

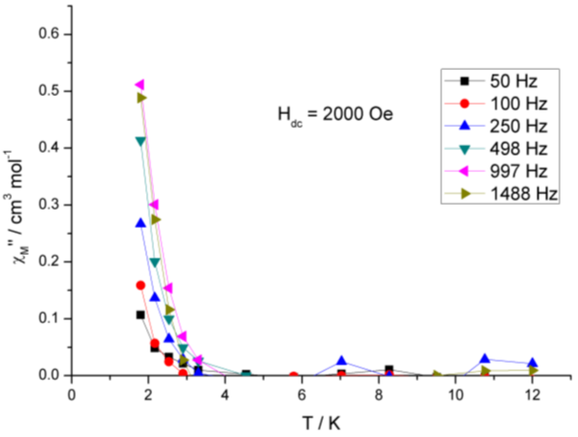

Supplementary Figure 3. Plot of χ_M'' versus T at the frequencies indicated for **1** with; (left)H_{dc} = 0 Oe and (right) H_{dc} = 2000 Oe. The negative value of χ_M'' are due to instrumental error for values near to zero.

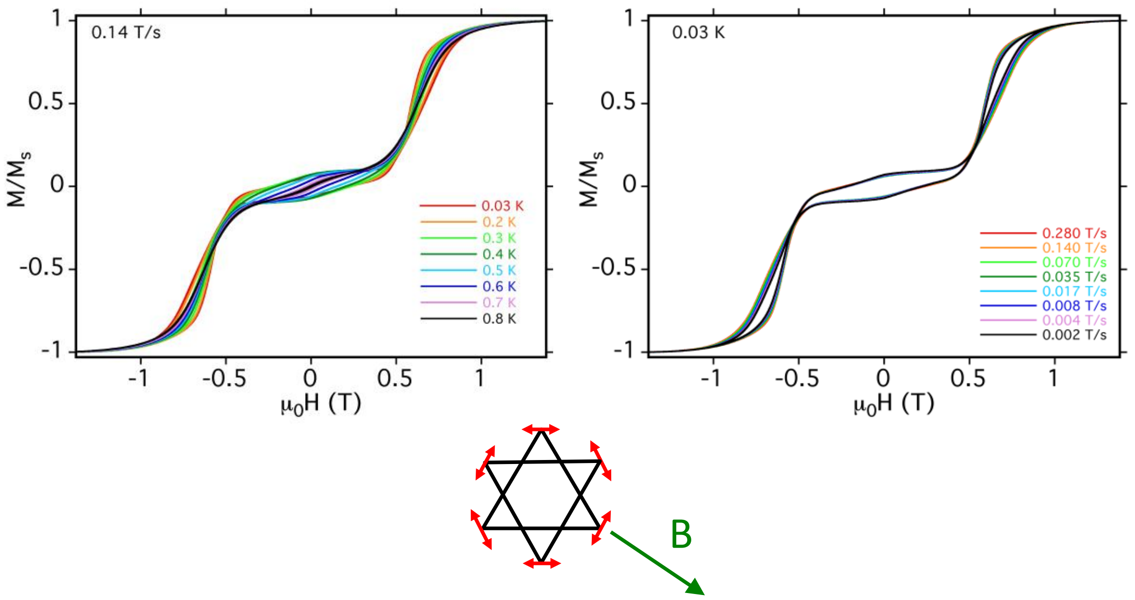

Supplementary Figure 4. Single-crystal magnetization (M) vs. applied field measurements (μ-squid) for complex **1** at (left) 0.03 K to 0.8 K with the scan rate of 0.14 Ts^{-1}; and (b) with different field sweep rates at 0.03 K. The orientation of the magnetic field is shown below.

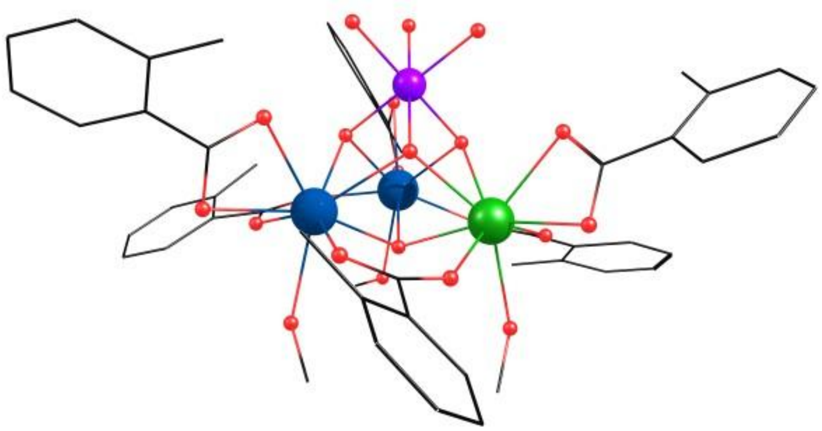

Supplementary Figure 5. The structure of the modelled Dy fragment employed for calculation (green, Dy^{III}; Dark blue, Lu^{III}, violet, Sc^{III}).

Supplementary Note 1. EPR simulation details.

Ab initio computed SINGLE_ANISO results of Dy1, Dy2 and Dy3 are employed as such along with the *J* values obtained from the simulation of susceptibility and magnetization data (see Supplementary Table 3 for the g-tensors and main text for the *J*s). We have employed only a $\{Dy_3Cr\}$ model with a pseudo S = 1/2 state for each Dy^{III} ion and a S = 3/2 state for Cr^{III} ion for the simulation. Calculations with full model employing $\{CrDy_6\}$ was not possible as the system size is very large. The (χ, ρ) angles for the Dy-Dy employed are (63.3,14.9), (53.2,1.2) and (60.2,7.2) for Dy1-Dy2, Dy2-Dy3 and Dy1-Dy3 pair respectively as obtained from the calculations. Similarly (χ, ρ) angles of (45.4,0.0), (60.5,0.0) and (4.7,0.0) is employed for Cr1-Dy1, Cr1-Dy2 and Cr1-Dy3 pairs respectively. A Gaussian Line width of 150 G is utilized. The exchange Hamiltonian employed is described in equation 2 in the main text, except that only $\{Dy_3Cr\}$ model is employed for the calculation.

Supplementary Note 2. Single-ion relaxation mechanism

A qualitative mechanism for the magnetic relaxation originating from the Dy1 site, obtained from the *ab initio* calculations, is shown in Supplementary Fig. 6 (see below). For all three Dy^{III} ions the ground state tunneling probability is computed to be small (for example 0.24 $x10^{-1}$ for Dy1) suggesting magnetization blockage occurs at the individual ion sites with a possible relaxation mechanism occurring via the first excited state through a thermally assisted quantum tunneling of the magnetization (TA-QTM) process. This qualitative mechanism yields information only about the possible QTM and TA-QTM processes while other possible relaxations such as the Raman process, deriving from intra/intermolecular interactions, nuclear-spins of the Dy^{III} ion and the ligand, spin-lattice relaxation, etc., are not taken in to consideration. Although this mechanism explains the presence of the low temperature out-of-phase signals at zero-field, the nature of the χ'' signals are similar when a 2000 Oe static dc field was applied and the computed barrier heights are much larger than that observed in the ac susceptibility measurements. This suggests that other factors are involved and the magnetic blocking does not arise simply from the single ion Dy^{III} anisotropy.

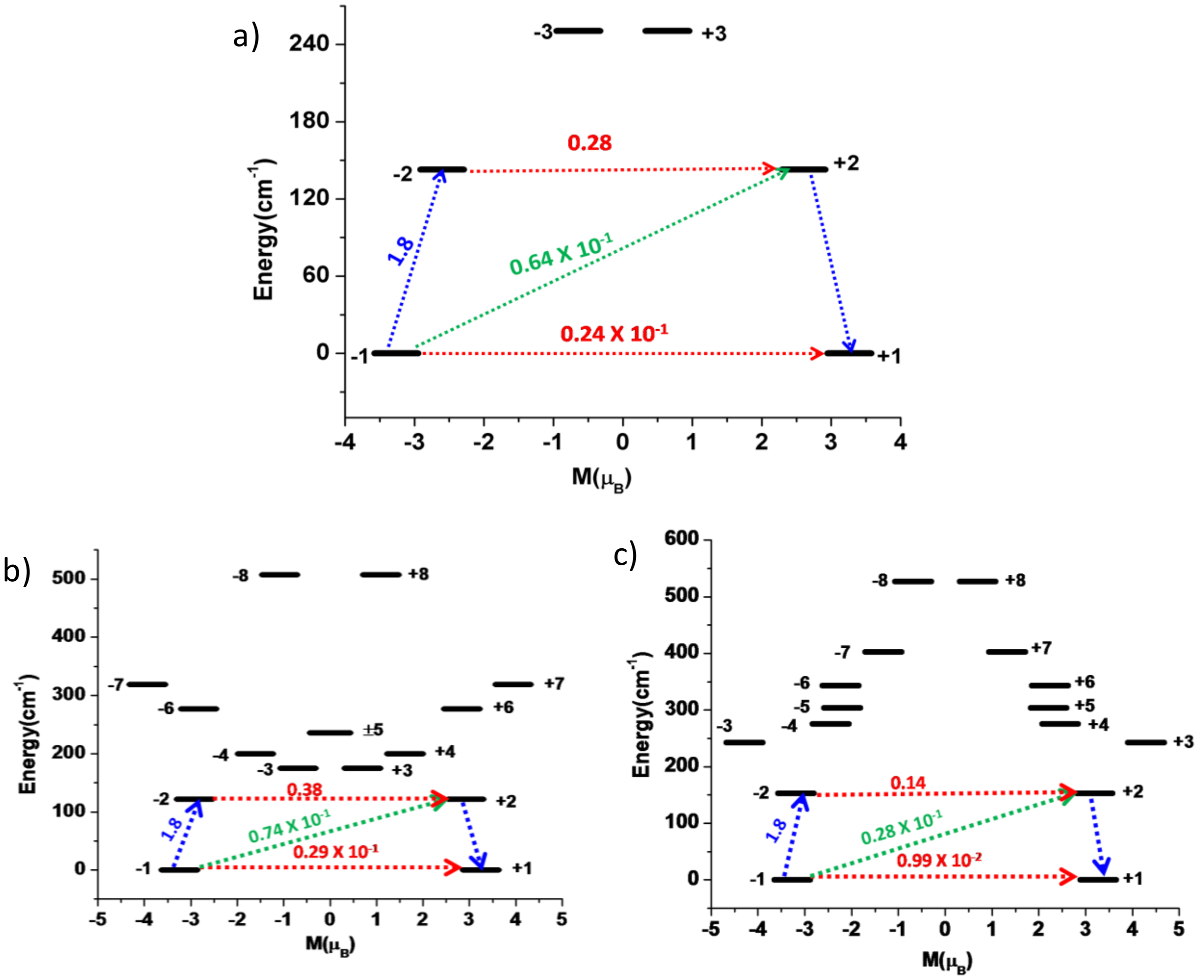

Supplementary Figure 6. The *ab initio* computed magnetization blocking barrier for a) the Dy1 site, b) the Dy2 site and c) the Dy3 site. The thick black line indicates the Kramers doublet (KDs) as a function of computed magnetic moment. The green/blue arrows show the possible pathway via Orbach/Raman relaxation. The dotted red lines represent the presence of QTM/TA-QTM between the connecting pairs. The numbers provided at each arrow are the mean absolute value for the corresponding matrix element of transition magnetic moment.

Supplementary note 3. ***How does our analysis of the experimental results exclude a non-toroidal arrangement?***

To probe the robustness of our conclusion i.e. that the ground state in our system is ferrotoroidically coupled, we varied one of the key results of our CASSCF-RASSI-SO calculations, which plays a crucial role in determining the ferrotoroidic ground state, namely the direction of the local anisotropy axes of the Dy ions, and used the resulting modified model to simulate the experimental magnetization. From our calculations the local anisotropy axes turn out to be almost exactly contained in the two triangles' planes, and directed along the local tangent to the wheel's circumference. To set up models that depart from this ab initio result, we generalized our exchange + dipolar coupling Hamiltonian introducing two angles: an angle η measuring the departure of the anisotropy axis from an in-plane configuration, and an angle ϕ measuring the departure of the in-plane projection of the anisotropy axis from a locally tangential direction. To comply with the D_{3d} pseudo-symmetry of the metal core of the complex, we demanded that the angle ϕ be the same for all Dy ions, while the angle η should have opposite signs for the two wheels, due to inversion symmetry.

We explored two significant scenarios departing from our parameter-free ab initio model, and reported the resulting powder magnetization curves obtained at 2 K in the figure below, together with the results of our parameter-free ab initio model (orange curve in the picture) and the experimental data points (blue data points in the picture):

(i) η = 30°, ϕ = 0°, *i.e.* a significant departure from in-plane tangential configuration of the magnetic axes, which will determine a significant out-of-plane magnetic moment for the Antiferrotoroidic (AFT) configuration only, but a zero out-of-plane magnetic moment for the Ferrotoroidic (FT) configuration. Such out-of-plane magnetic component of the AFT state will also be coupled antiferromagnetically to the Cr magnetic moment, thus stabilizing the AFT with respect to the non-magnetic FT state. For η = 30°, the appearance of a significant anisotropic out-of-plane magnetic moment in the AFT state, determines a strong Cr-Dy_6 antiferromagnetic stabilisation energy contribution which makes the AFT configuration the ground state, and the FT state the first excited state. However, the powder magnetization we calculate in this scenario is reported in the picture below (green curve), and evidently it does not match the experimental data, which instead support our finding that at low field the only source of magnetic response comes from the Cr ion. Any additional (anisotropic) magnetism from the Dy-triangles would make the low-field magnetization steeper than what is observed

experimentally, which supports our finding of the FT configuration (implying a zero magnetic moment on the wheels) as the ground state.

(ii) $\eta = 0°$, $\phi = 90°$, *i.e.* the axes are still perfectly in-plane (contained in the planes defined by the two triangles), but they are now directed radially instead of tangentially to the triangle's circumference. In such configuration it is still possible to achieve a non-magnetic non-collinear ground state on the Dy wheels, for which the magnetism solely arises from isotropic paramagnetic Cr. However, as first pointed out by some of us[1] in radially configured anisotropy axes, pure dipolar coupling does not favour such non-collinear non-magnetic configuration of the Dy magnetic moments, and favours instead a large in-plane magnetic moment in the ground state of each wheel. Furthermore, antiferromagnetic coupling to Cr ion determines the ferromagnetically coupled state (i.e. the state where the in-plane magnetic moments of the two Dy triangles lie parallel to each other) as the ground state, so that in fact adopting a radial instead of a tangential configuration of the magnetic axes leads to a strongly magnetic and strongly in-plane anisotropic ground state, while the states in which the two triangles have zero magnetic moment are the highest in energy. This simple rotation of the Dy anisotropy axes, still compliant with the system's pseudo-symmetry, and still allowing for the existence of non-magnetic states on the Dy triangular wheels, leads to a dramatically different exchange and dipolar coupled spectrum for $CrDy_6$ complex from the FT ground state predicted by our parameter-free model. However, due to the large and strongly anisotropic magnetism arising from this scenario, the low-field powder magnetization is in fact dramatically different from that experimentally observed, as can be seen from its plot in the figure below (red curve). This model also suggests that the ab initio calculations accurately reproduce the direction of the local anisotropy axes as in-plane tangential, thus stabilizing a ferrotoroidic ground state in which the magnetism solely arises from the Cr spin.

We believe that this extended model, together with the fact that our proposed model is parameter-free, only relying on experimental information (i.e. geometry of complex) and ab initio calculations, provide strong evidence that the ground state of the title compound CrDy6 is indeed ferrotoroidically coupled.

Furthermore in the new revised version we introduce a substantial extension of our discussion of the dynamics of the magnetization in this system as observed from the single crystal magnetization experiments also introducing a theoretical model of the spin dynamics based on our model Hamiltonian which allows us to simulate and reproduce the zero-field

hysteretic magnetic response observed in the experiments This point is further discussed below but we believe it provides further evidence for the validity of our conclusions

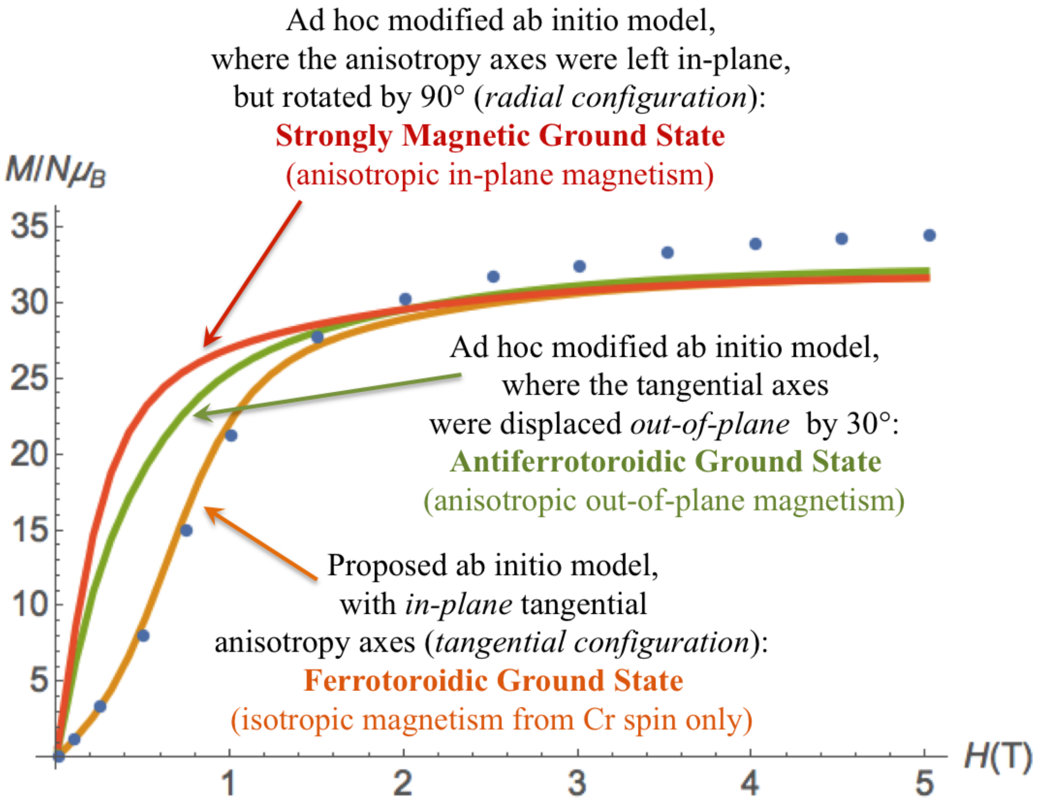

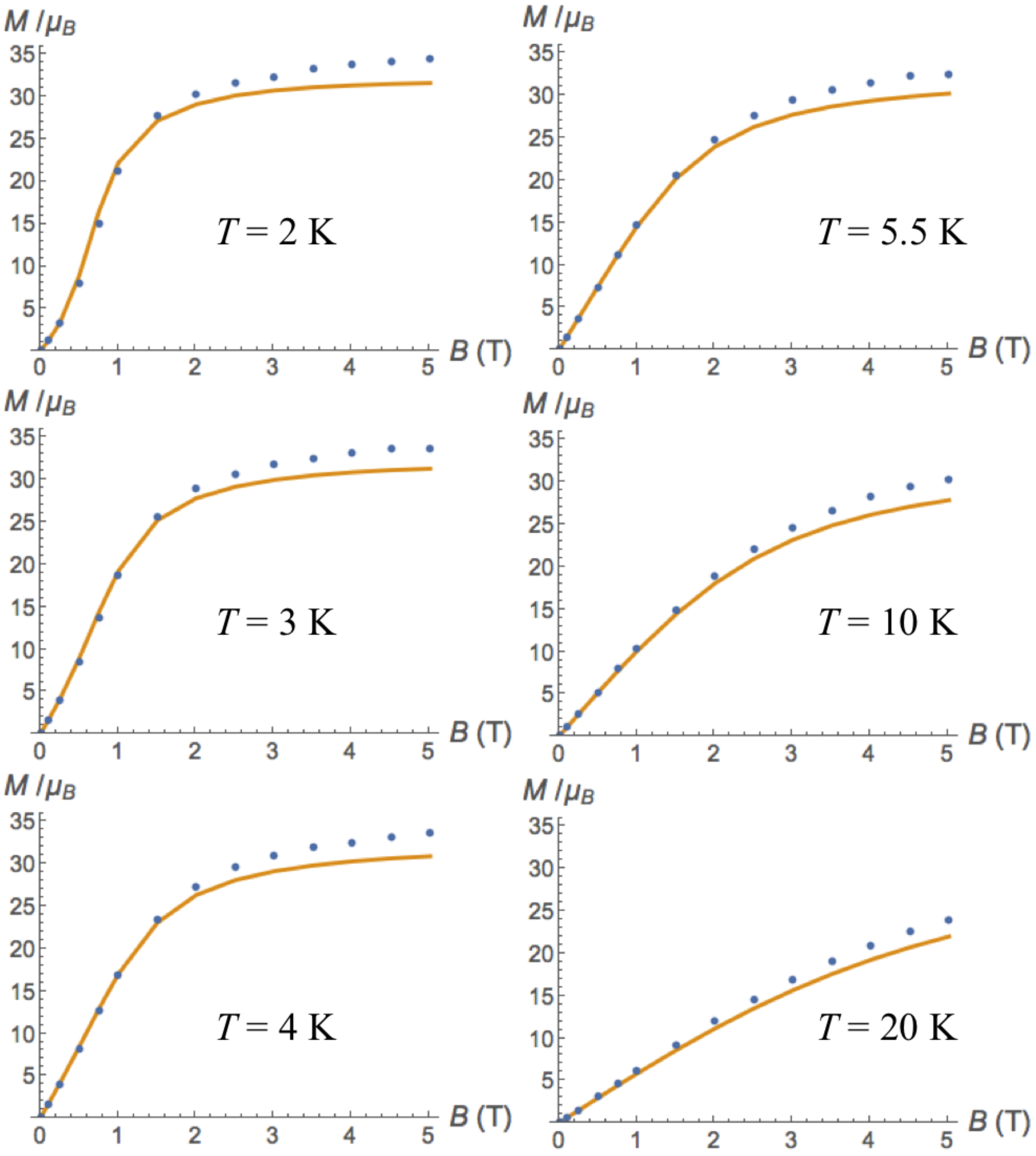

Supplementary Figure 7. Measured *M* vs *H* for complex **1** (dots) and the theoretical *M* vs *H* plot obtained from the model described in the text (solid line).

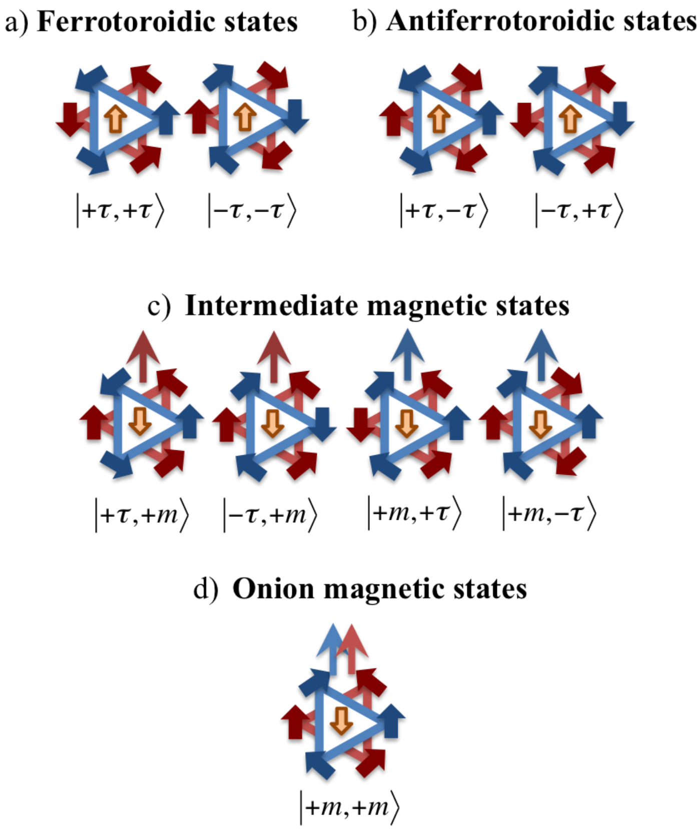

Supplementary Figure 8. Graphical representation of the states of $CrDy_6$ that are stabilized by a magnetic field contained in the plane of the figure, and oriented from bottom to top. The blue (red) arrows at the vertices of the top (bottom) triangle atomic positions represent the local Dy^{3+} magnetic moment in that state, while the central yellow arrow represents the Cr magnetic moment. The blue (red) thin arrows lying above the molecular system in panels c) and d) represent the direction of a total magnetic moment of ~20 mB arising from the sum of the Dy^{3+} atomic magnetic moments belonging to the top (bottom) triangle. Note that only in the ferrotoroidic states in panel a) and antiferrotoroidic states in panel b) the Cr magnetic moment is oriented along the field, while in the other states due to Dy-Cr antiferromagnetic coupling the Cr magnetic moment is oriented opposite to the magnetic field.

Supplementary Note 4. Comparison of toroidal coupling in $CrDy_6$ and other Dy_6 complexes.

The coupling of two or more triangular Dy_3 rings, each stabilizing a toroidal moment in their ground state, into a structure presenting new collective magnetic properties has been previously explored in three important works:

1) The earliest work on a Dy_6 cluster (**Dy_6-1**) composed of magnetically coupled triangular subunits is that by Hewitt et al.,[2] who achieved a **Dy_6-1** SMM composed of two co-parallel but non co-planar triangles, each characterized by uniaxial Dy ions with anisotropy axes quasi-tangentially arranged around the triangles' circumference, and characterized by an inter-triangle antiferromagnetic coupling via two neighbouring vertices. As already pointed out by Lin et al.[3] the work of Hewitt et al.,[2] nicely shows how coupling between toroidal states can offer new mechanisms to enhance slow magnetic relaxation, although due to the particular geometry of coupling achieved in that system, the total toroidal moment of **Dy_6-1** is not maximized by such coupling, as the antiferromagnetic inter-wheel interaction is such as to cancel the contribution from the Dy magnetic moment of the two coupled vertices to the overall vortex magnetisation. In the system we present here the ferrotoroidic ground state implies on the other hand a maximization of the toroidal moment achievable by six Dy ions.

The nature of the low-energy states in **Dy_6-1** were indeed graphically represented in the supplementary information file in terms of con-rotating and counter-rotating toroidal moments on the two triangular subunits, although the ferrotoroidic and/or antiferrotoroidic nature of such states was not explicitly discussed, or even mentioned in the main text of the paper. Given that ferrotoroidic and antiferrotoroidic states are non-magnetic (or weakly magnetic due to deviation from co-planarity of the Dy anisotropy axes), hence they make little contribution to the magnetic response of the system, at this stage where no direct experimental technique can easily probe the toroidal character and relative energy ordering of con-rotating and counter-rotating toroidal states, it is essential to minimize the source of free parameters in the theoretical models used to characterized them. In this respect, the fact that the Hamiltonian used by Hewitt et al. in their work contains four fitting parameters, and neglects dipolar coupling, suggests that more detailed investigations of **Dy_6-1** are necessary to make definitive statements about the ferrotoroidic nature of its ground state. We note here that the model Hamiltonian used here to simulate the magnetic data for $CrDy_6$ includes

dipolar coupling (which in fact is shown to dominate the resulting energy spectrum), and contains no fitting parameter.

2) Lin et al.,[3] have presented an interesting study of a **Dy_6-2** cluster that can be viewed as two very closely spaced Dy_3 triangular units with two edges, one from each triangle, directly facing each other. The system is not exactly co-planar, but displays a 29º dihedral angle between the two triangles' planes. In that work the magnetic coupling is modeled including both dipolar and exchange coupling, and the number of free parameters is greatly reduced with respect to the Hamiltonian reported by Hewitt et al., i.e. Lin et al. only use one fitting parameter.

We note however that there are a few features of the Hamiltonian used in that paper that would seem to need further testing before the conclusions drawn about the nature of the ground state be unambiguously confirmed. First of all, we note that the choice of a single exchange coupling parameter, especially given that this is a fitting parameter not derived from a theoretical model or an ab initio calculation, can be in principle criticized. In particular, we note that while antiferromagnetic coupling between ions belonging to the same triangle is known to stabilize a toroidal moment, given the geometry of **Dy_6-2**, antiferromagnetic coupling between nearest neighbor ions on different triangles (e.g. Dy_1 and Dy_2 in Fig. S1 of that paper) will in fact stabilize counter-rotating toroidal states on different triangles, hence an antiferrotoroidic ground state. Given that the distance between Dy_1 and Dy_2 in Fig. S1 is shorter (3.34 Å) than any intra-triangular Dy-Dy distance (3.39Å, 3.51Å, 3.54Å), it could be argued that a stronger inter-molecular antiferromagnetic exchange between such ions could flip the energetic order of the con-rotating and counter-rotating coupled-toroidal states. On the other hand, an antiferromagnetic diagonal interaction (e.g. between Dy_1-Dy_3 in Fig. S1 of that paper) will indeed stabilize a con-rotating toroidal configuration, but the distance between the Dy ions is in CrDy6 (**1**) much longer, thus weakening such interaction (Dy_1-Dy_3 distance is 4.7Å). Such important competing effects are clearly not captured by a single fitting parameter, and these issues are not discussed by Lin et al.[3] (We present alternative calculations on **Dy_6-2** and these are discussed below).

Aside from the details of the Hamiltonian parameterisation, assuming that the arrangement of local Dy magnetic moments in the ground state of **Dy_6-2** leads to a maximization of the overall toroidal moment of the molecule, to characterize such ground state as a ferrotoroidically-coupled state still seems somewhat problematic on the grounds of two

issues, which are in fact related to each other: (i) the triangle-triangle distance is shorter than two of the intra-triangle's Dy-Dy distances, so that the detailed connectivity of the central Dy_4 skewed rectangle is bound to play a central role in determining the final magnetic configuration of the system. This fact poses questions as to whether it is possible to identify the large toroidal moment in the ground state as resulting from the coupling of two well-defined separate toroidal subunits, or rather as an overall Dy_6 perimeter toroidal arrangement of magnetic moments resulting from the detailed molecular connectivity of this cluster, that cannot be simply analysed solely in terms of separate triangular subunits (ii) as a consequence, assuming the model with a single exchange coupling parameter presented by Lin et al.[3] is valid, the excited putative antiferrotoroidic state in which the toroidal moment on one triangle is counter-rotating with respect to the toroidal moment on the second triangle lies in fact at a relatively high energy, at the same energy in fact as that of purely magnetic excitations not determined by the simultaneous flipping of the spins on all Dy belonging to one triangular subunit. The lack of low-lying antiferrotoroidic excitations (or rather, the effective energetic equivalence of toroidal excitations and magnetic excitations), renders this system a somewhat less clear cut case of a well-defined magnetic coupling between separate toroidal subunits such as the case presented in this paper, albeit an interesting example of how to achieve a large toroidal moment in the ground state.

3) Novitchi et al.[4] reported the first example of exchange coupling between toroidal moments in a chiral heterometallic Cu^{II}/Dy^{III} polymer, built from alternating Dy_3 SMM building blocks. The ground state of such system has been found to be antiferrotoroidic, and it is argued there that the ferrotoroidic first excited state, having a magnetic component, can be stabilized by magnetic field, so that in a magnetic field applied in the appropriate direction the ground state would become ferrotoroidic (but not degenerate).

These previous works, and a tutorial review of these works by Tang et al.,[5] unveil crucial information concerning microscopic pathways to the coupling of molecular toroidal moments, in addition to discussing how to harness the resulting states to enhance SMM slow relaxation properties (**Dy_6-1**, Hewitt et al.), and to enhance the overall toroidal moment of a single molecule (**Dy_6-2,** Lin et al.).

Nevertheless, to the best of our knowledge, the $CrDy_6$(**1**) system presented here, according to our parameters-free model, provides the first example of a well-defined ferrotoroidic ground state resulting from the coupling of two separate toroidal subunits, maximizing the total

toroidal moment, and characterized by low-lying pure toroidal excitations to antiferrotoroidic states (counter-rotating toroidal moments resulting in zero toroidal moment), well separated from higher-energy magnetic excitations.

Alternative calculations on Dy_6-2 complex: To test alternative scenarios for **Dy_6-2** we have set up an approximate model Hamiltonian in which the two triangles are considered equilateral using average experimental bond distances, the 29º dihedral angle between the two triangles' planes is explicitly taken into consideration, and the deviation from co-planarity of the Dy anisotropy axes is also included in the model using the data reported in that paper. Dipolar coupling is explicitly included in the model as is exchange coupling (see equation 1 in the main manuscript for the dipolar Hamiltonian). However, we introduce here additional exchange coupling constants to differentiate between intra-triangle coupling, and the two independent inter-triangle coupling pathways. To illustrate our point, with no ambition to find optimal fitting parameters in a model that would become quickly over parameterised, we used our Hamiltonian to simulate the experimental data presented by Lin et al. (digitalized from paper), both (i) using a single exchange coupling parameter J_{Lin} as reported by Lin et al. thus reproducing their results, and (ii) by setting to zero the diagonal exchange coupling between the distant cross-coupled ions 4.7Å apart ($J_{\mathrm{diag}}=0$), and using two different constants, one describing intramolecular coupling ($J_{\mathrm{intra}} = 1.8\ J_{\mathrm{Lin}}$) and intermolecular coupling ($J_{\mathrm{inter}} = 1.5\ J_{\mathrm{Lin}}$). The resulting spectra and states are reported in Supplementary Fig. 9, together with the simulation of the low-temperature (2 K) powder magnetization compared for the two models (orange curve describes the model of Lin et al, the green curve is associated to the parameters discussed above). It can be seen that the simulation of the experimental data is hardly changed in the two settings, but for a single parameter the ground state consists of con-rotating toroidal states separated by a large energy gap from the counter-rotating toroidal state (transition indicated with a blue arrow in Supplementary Fig. 9), almost at the same energy as the first magnetic excitation (transition indicated with a red arrow in Supplementary Fig. 9), while with the new parameters tried here the con-rotating and counter-rotating energy ordering is inverted, and the toroidal excitation is much smaller than the magnetic excitation.

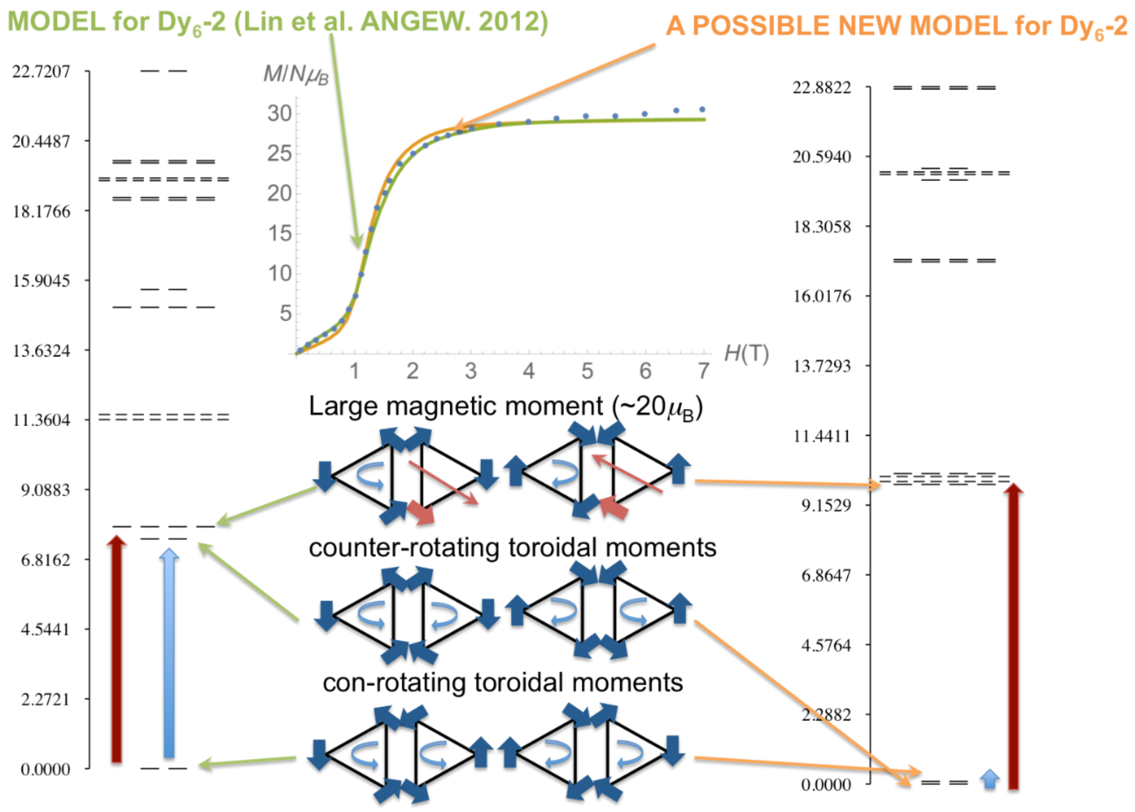

Supplementary Figure 9. The energy spectra (cm^{-1}) and schematic representation of the Dy ion magnetic moment arrangement in the low-lying collective magnetic states for the the molecule **Dy$_6$-2**, modeled using the Hamiltonian reported with parameters (i) $J_{intra} = J_{inter} = J_{diago} = J_{Lin} = -0.2349$ cm^{-1}, i.e. essentially the model reported in Lin et al. (spectrum on the left), and (ii) $J_{intra} = 1.8\ J_{Lin}$, $J_{inter} = 1.5\ J_{Lin}$, $J_{diago} = 0.0$ (spectrum on the right). The transitions between ground and lowest lying excited states are indicated with a red (blue) arrow if the excitation is magnetic (toroidal) in nature. In the top part of the figure a simulation of the powder magnetization at temperature $T = 1.9$ K is reported, obtained using either model (i) i.e. the Lin et al. one-parameter model (green curve), or the three-exchange parameters model (ii) discussed above (orange curve), together with the experimental data (blue data points) digitalized from Ref. Lin et al.[3]

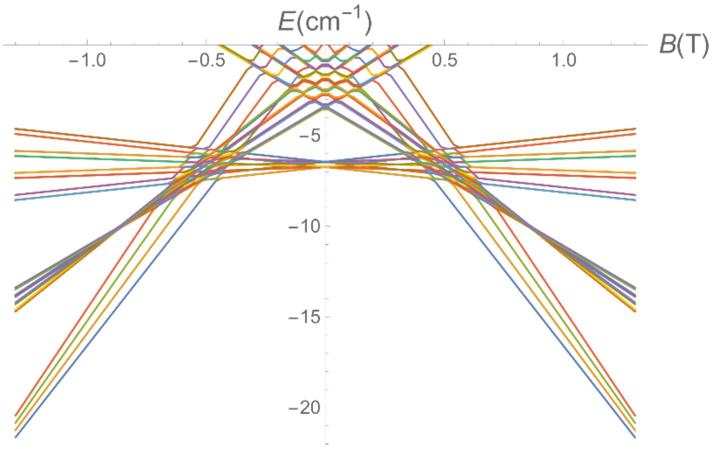

Supplementary Figure 10. Energy levels of $CrDy_6$ as function of magnetic field (Zeeman spectrum) computed using the parameter-free theoretical model presented in the text, but retaining only those 56 states out of 256 that have magnetic anisotropy axes oriented along the *y*-axis in Figure 6 (easy-axis), along which the sweeping field of the single-crystal experiment is oriented. These are the 56 states we have retained in our dynamical model Equation (11).

Supplementary Note 5. Further analysis of the theoretical dynamical magnetization

To further analyse our simulation of the dynamical magnetization, we report the plots of the contributions to $M(t) = \mathrm{Tr}[\sigma(t)M]$ arising from the ferrotoroidic and antiferrotoroidic states (Supplementary Figure 11(b)), and from the intermediate magnetic states and the onion states (Supplementary Figure 11(c)). It can be seen that the hysteresis loop about the zero field region is not produced by the free fluctuating paramagnetic Cr spin (in zero field the magnetization of the FT and AFT states in Supplementary Figure 11(b) is zero), but, as evident from Supplementary Figure 11(c), the zero-field hysteresis loop arises from the lagging in the depletion of the non-equilibrium populations of the excited intermediate states predominantly, with contributions from the onion magnetic states as well. Note also that the contribution of FT states to the dynamics is visibly much larger than that of the AFT states.

Finally, we note that, due to the choice of coupling constants, the dynamics portrayed in Supplementary Figure 11 is dominated by the 1-flip transitions, as evidenced by the fact that the magnetization at high fields is dominated by the excited intermediate states (see Supplementary Fig. 11(c)). If we now solve Equation (8)/ Equation (11) for a slightly different set of parameters, still preserving the proposed hierarchy, but using faster 2-flip transitions, so that $\Gamma_{Cr} = 3 \times 10^4$ Hz/(cm^{-1})^3>>$\Gamma_1 = 3.33 \times 10^{-7} \times \Gamma_{Cr} > \Gamma_2 = 10^{-2} \times \Gamma_1$, and γ_{Cr} = 4×10^{15} Hz^2>>$\gamma_1 = 2 \times 10^{12}$ Hz^2>$\gamma_2 = 10^{-1}\times\gamma_1$, we obtain a hysteretic magnetization reported in Supplementary Figure 12(a), which still reproduces the zero-field hysteresis loop, with an

almost closed hysteresis at high fields, but now dominated by the onion states at high fields (see Supplementary Figure 12(c)), and displaying a sizeable contribution from AFT states at low-fields, almost as large as that of the FT states (see Supplementary Figure 12(b)), but otherwise not changing the main conclusions drawn above.

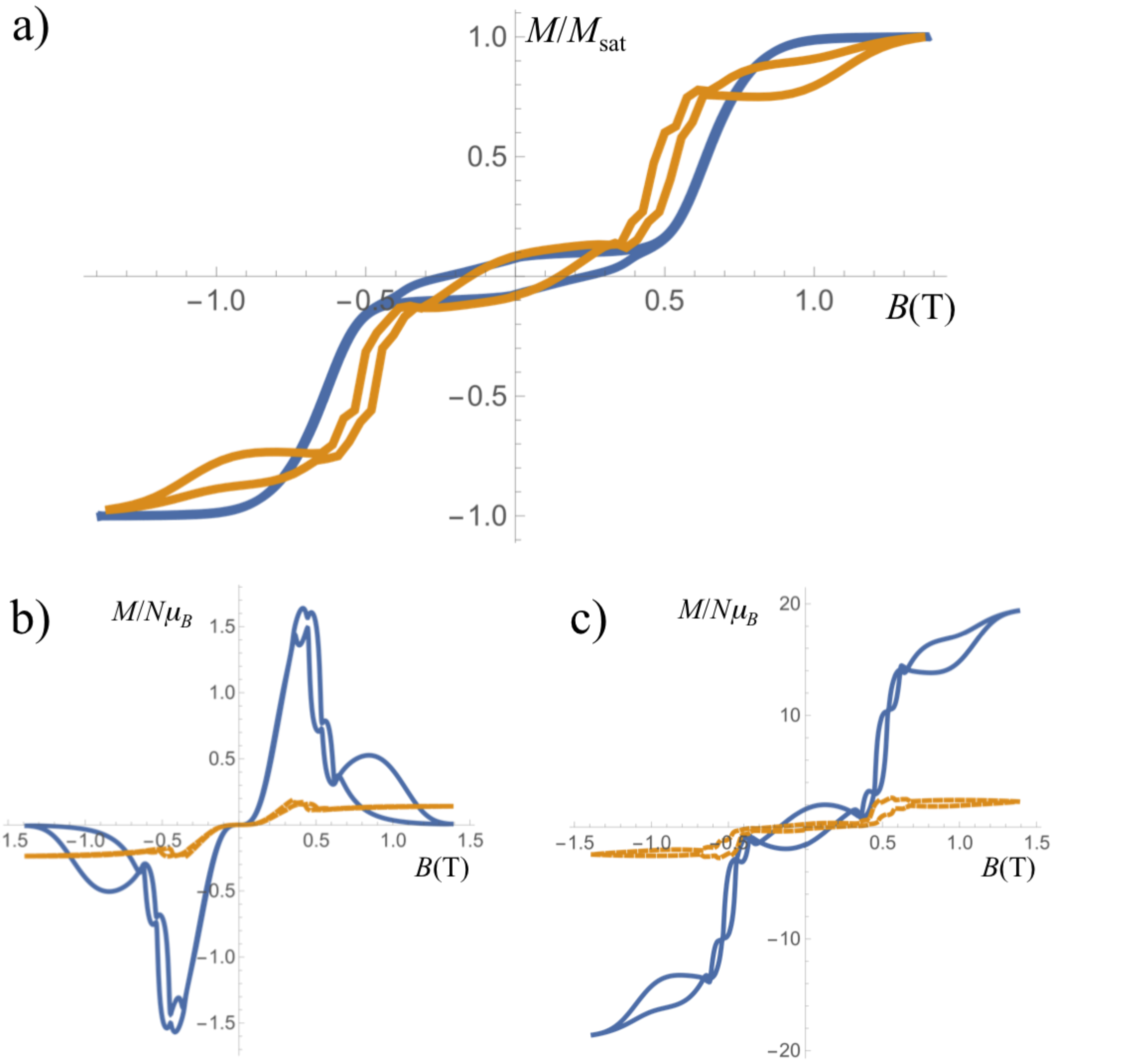

Supplementary Figure 11. a) Single-crystal experimental magnetization (blue curve) measured at T = 0.03 K and a sweep rate of 0.1T/s for a magnetic field oriented parallel to the triangles' planes and along the easy-axis (y axis in Figure 6), superimposed to the simulated dynamical magnetization at the same temperature, sweep rate and field orientation, by solving Equation (11)/ Equation (8) on the basis of 56 out of the 256 states obtained from our model and reported in Supplementary Figure 8, for the following numerical values of the transition rates appearing in the equations: $\Gamma_{Cr} = 10^5$ Hz/(cm^{-1})^3$>>\Gamma_1 = 10^{-7}\times\Gamma_{Cr}>\Gamma_2 = 10^{-3}\times\Gamma_1$, and $\gamma_{Cr} = 10^{16}$ Hz^2$>>\gamma_1 = 10^{12}$ Hz^2$>\gamma_2 = 10^{-3}\times\gamma_1$; b) Contribution to the total simulated dynamical magnetisation reported in panel a) from the ferrotoroidic (blue solid line) and antiferrotoroidic (orange dashed line); c) Contribution to the total simulated dynamical

magnetisation reported in panel a) from the intermediated magnetic states (blue solid line) and onion states (orange dashed line).

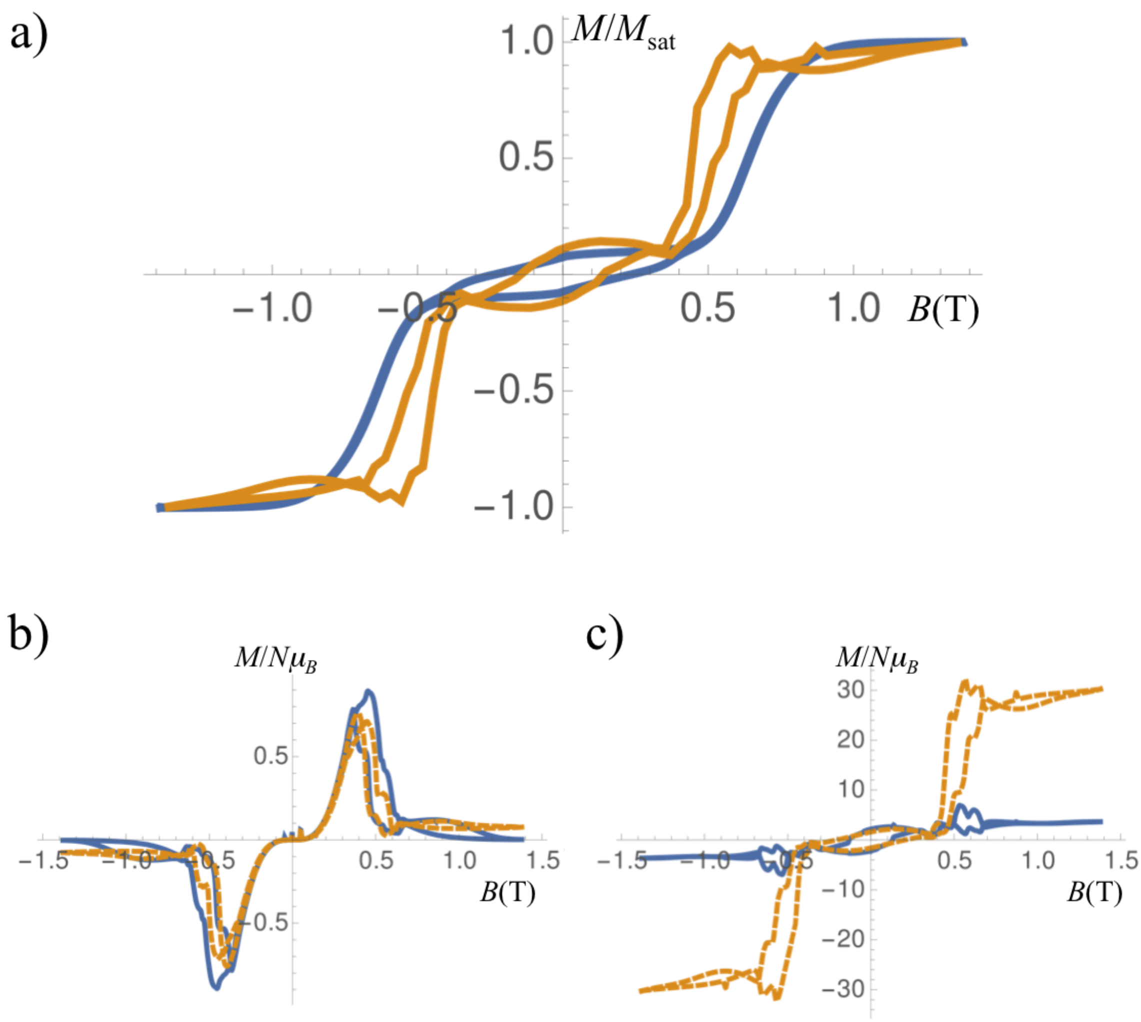

Supplementary Figure 12. a) Single-crystal experimental magnetization (blue curve) measured at T = 0.03K and a sweep rate of 0.1T/s for a magnetic field oriented parallel to the triangles' planes and along the easy-axis, superimposed on the simulated dynamical magnetization at the same temperature, sweep rate and field orientation, by solving Equation (11) on the basis of 56 out of the 256 states obtained from our model, for the following values of the transition rates: $\Gamma_{Cr} = 3 \times 10^4$ Hz/(cm^{-1})^3$>>\Gamma_1$ = 3.33 $\times 10^{-7} \times \Gamma_{Cr} > \Gamma_2 = 10^{-2} \times \Gamma_1$, and $\gamma_{Cr} = 4\times10^{15}$ Hz^2$>>\gamma_1 = 2 \times 10^{12}$ Hz^2$>\gamma_2 = 10^{-1}\times\gamma_1$; b) Contribution to the total simulated dynamical magnetisation reported in panel a) from the ferrotoroidic (blue solid line) and antiferrotoroidic (orange dashed line); c) Contribution to the total simulated dynamical magnetisation reported in panel a) from the intermediated magnetic states (blue solid line) and onion states (orange dashed line).

Supplementary References

1. Giansiracusa, M. J. *et al.* Carbonate-Bridged Lanthanoid Triangles: Single-Molecule Magnet Behavior, Inelastic Neutron Scattering, and Ab Initio Studies. *Inorg. Chem.* **55**, 5201-5214 (2016).
2. Hewitt, I. J. *et al.* Coupling Dy_3 Triangles Enhances Their Slow Magnetic Relaxation. *Angew. Chem. Int. Ed.* **49**, 6352-6356 (2010).
3. Lin, S.-Y. *et al.* Coupling Dy_3 Triangles to Maximize the Toroidal Moment. *Angew. Chem. Int. Ed.* **51**, 12767-12771 (2012).
4. Novitchi, G. *et al.* Heterometallic Cu^{II}/Dy^{III} 1D chiral polymers: chirogenesis and exchange coupling of toroidal moments in trinuclear Dy_3 single molecule magnets. *Chem. Sci.* **3**, 1169-1176 (2012).
5. Ungur, L., Lin, S.-Y., Tang, J. & Chibotaru, L. F. Single-molecule toroics in Ising-type lanthanide molecular clusters. *Chem. Soc. Rev.* **43**, 6894-6905 (2014).
